# Neuroendocrine breast carcinoma

**DOI:** 10.4322/acr.2024.484

**Published:** 2024-03-21

**Authors:** Laura Pratas Guerra, Joana Simões, Diogo Carvalho Sá, José Polónia, António Araújo

**Affiliations:** 1 Unidade Local de Saúde de Santo António, Serviço de Oncologia Médica, Porto, Portugal; 2 Unidade Local de Saúde de Santo António, Serviço de Anatomia Patológica, Porto, Portugal; 3 Unidade Local de Saúde de Santo António, Unidade de Patologia Mamária, Porto, Portugal; 4 Universidade do Porto, Instituto de Ciências Biomédicas Abel Salazar, Unidade de Investigação em Oncologia, Unidade Multidisciplinar de Investigação Biomédica, Porto, Portugal

**Keywords:** Carcinoma, Neuroendocrine, Breast Cancer, Pathology

## Abstract

Neuroendocrine breast cancer (NEBC) is a rare and heterogeneous entity. It most commonly presents a luminal phenotype and a worse prognosis. When diagnosed in an advanced stage, metastasis from another neuroendocrine tumor should be excluded.

This case features a premenopausal woman with an oligometastatic breast large cell neuroendocrine carcinoma, estrogen receptor (ER) positive, and human epidermal growth factor receptor 2 (HER2) negative. Since the patient was very symptomatic at the presentation of the disease, chemotherapy was started. Complete radiological response of the metastatic disease was achieved, and the patient was then submitted to radical breast surgery and bilateral oophorectomy. She subsequently underwent radiation therapy. Since then and to date, she has been under endocrine therapy (ET) and a CDK4/6 inhibitor (CDK4/6i), with no evidence of malignant disease.

Evidence to guide the choice of treatment for these tumors is currently scarce. In cases with oligometastatic disease, radical treatment should be considered. Given that this entity is rare, its reporting should be encouraged.

## INTRODUCTION

Neuroendocrine breast cancer (NEBC) is a rare and heterogeneous entity, accounting for 0,1 to 5% of all invasive breast carcinomas.^1,2^ These tumors are more commonly diagnosed in women between the sixth and seventh decade of life.^2^ According to the World Health Organization classification, primary neuroendocrine neoplasms can be well-differentiated, being classified as neuroendocrine tumors (carcinoid-like and atypical carcinoid-like) or poorly differentiated, being classified as neuroendocrine carcinomas (small cell neuroendocrine carcinoma and large cell neuroendocrine carcinoma (LCNEC)).^3^ The diagnosis is established by the presence of cells’ neuroendocrine architecture and the expression of several markers, such as chromogranin A (CgA) and synaptophysin (Syn).^4^

More frequently, NEBC exhibits a luminal phenotype (A or B), being hormone receptors (HR) positive and HER2 negative.^1^ However, most recent studies have shown a poorer outcome for these tumors when compared to those without neuroendocrine differentiation.^2^ They do not usually manifest as carcinoid syndrome.^1^

NEBC can spread to various sites, mostly to bones and the liver.^2^ Metastasis from another primary neuroendocrine tumor should always be excluded as a differential diagnosis using whole-body computed tomography (CT) or positron emission tomography (PET) scans. The latter should be a fluorodeoxyglucose-PET (FDG-PET) if the tumor is poorly differentiated and a gallium-PET if the tumor is well differentiated. 

Evidence shows that the NEBC grade and Ki67 percentage are prognostic factors affecting disease-free survival and that age and ER status are prognostic factors impacting overall survival.^5^

Currently, there is a lack of evidence to guide the choice of treatment for NEBC, whether in the early or advanced stage. Surgery remains the main option with ET.^3^ Chemotherapy is usually reserved for tumors with a high risk of recurrence, and CDK4/6i, although lacking evidence from prospective clinical trials, have been used with favorable responses, as reported by a few clinical cases.^3,6^

## CASE REPORT

This clinical case features a 46-year-old premenopausal woman, Eastern Cooperative Oncology Group Performance Status of 1, with no relevant medical history, including no familiar history of cancer. She was nulliparous and had never used oral contraceptives.

On self-examination, she noticed a lump in her right breast and was very symptomatic, complaining of pain in her right arm. On clinical examination, there was no cervical or supraclavicular adenopathy. She had a nodular mass measuring 40 x 20 mm in the transition of the inferior quadrants of the right breast, as well as thickening of adjacent skin without ulceration. Another nodule was present in the lower outer quadrant of the right breast, measuring 20 mm, and axillary lymphadenopathy, measuring 40 x 30 mm.

A breast ultrasound was performed, showing several nodules in the right breast, suggestive of multicentric breast cancer (BI-RADS 5). The biggest nodule, measured 46 x 21 mm, was located in the retroareolar region with contact with the adjacent skin. The second biggest nodule was located in the transition of the inferior quadrants, measuring 21 x 13 mm, and was also in contact with the adjacent skin. The ultrasound also identified several right axillary enlarged lymph nodes, the largest measuring 41 x 33 mm and another measuring 21 x 15 mm. There were no abnormalities in the contra-lateral breast or in the left axilla.

**Figure 1:**
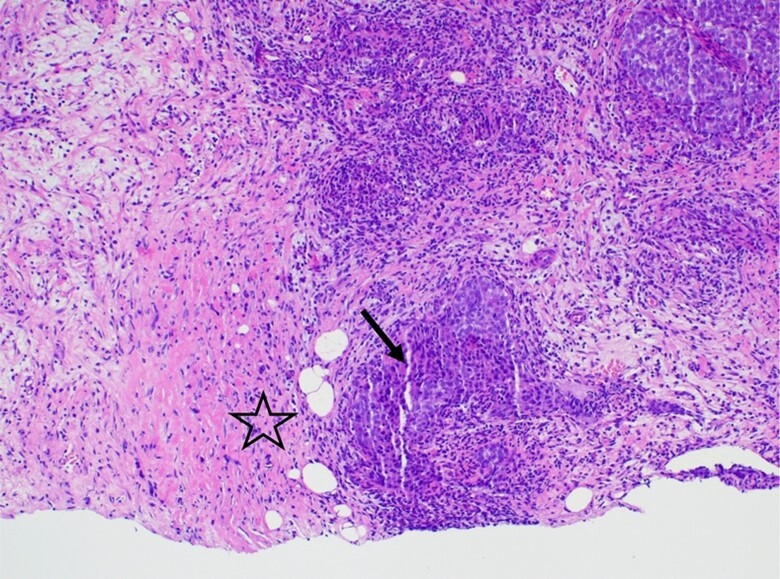
Photomicrographs of the breast and tumor. The interface between tumor (arrow) and breast parenchyma (star) (H&E, 100x).

**Figure 2:**
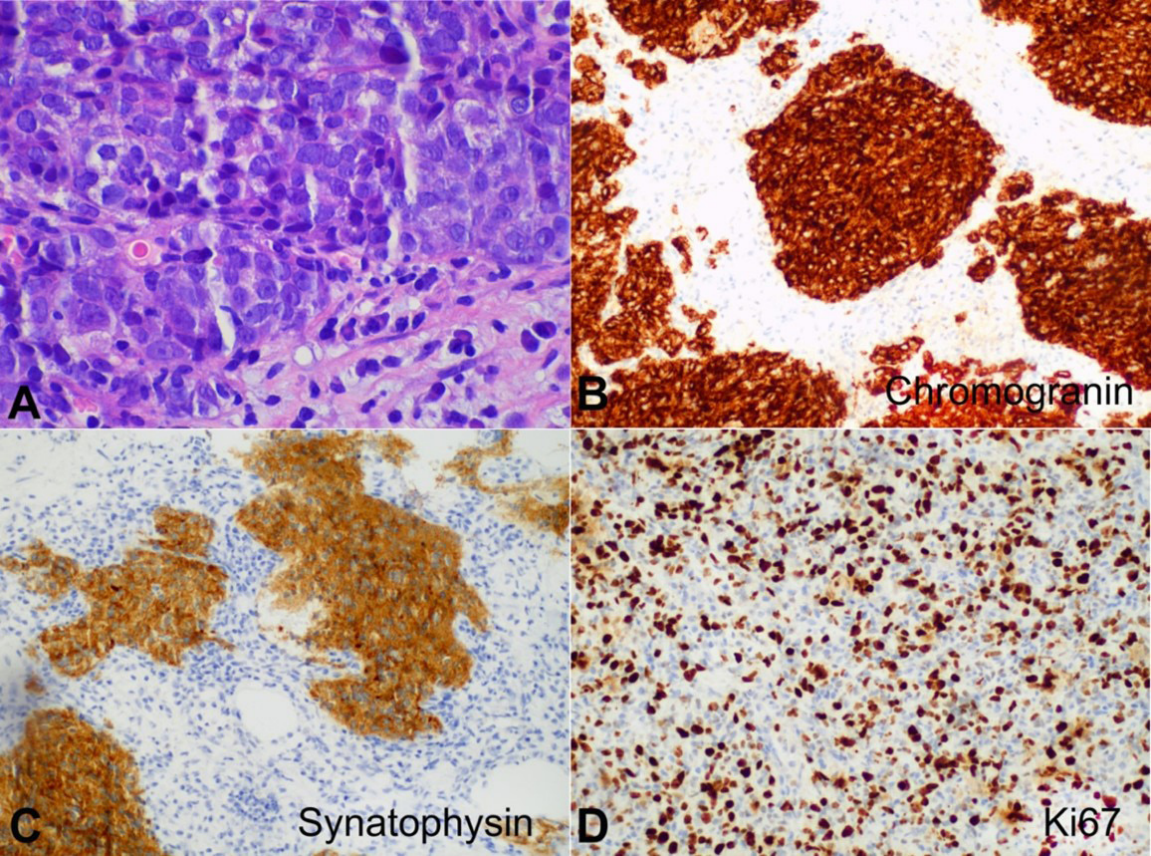
Photomicrographs of the tumor. **A** - Large cell neuroendocrine carcinoma composed of nests of large cells with prominent nucleoli (H&E, 600x); **B** - positive reaction to Chromogranin (200x); **C** - positive reaction to Synaptophysin (200x); **D** - Ki-67 showed a proliferation index of 50%.

A core needle biopsy was performed, revealing an LCNEC composed of nests of large cells (Figure 1) with prominent nucleoli showing expression of CgA and Syn, Ki67 50%, with lymphovascular invasion, ER 70%, progesterone receptors 1%, HER2 negative (score 1+ by immunohistochemistry), E-cadherin positive, CK19 positive and GATA3 positive (Figure 2). Carcinoma *in situ* was not found. The axillary biopsy showed a malignant neoplasm with the same characteristics. Genetic testing of 18 genes (including BRCA 1/2) found no pathogenic variants.

An FDG-PET scan showed a multifocal malignant lesion with a high metabolic index (maximum standardized uptake value (SUVmax) 9.4) in the right breast with local cutaneous invasion, as well as right axillary lymphatic metastasis (SUVmax 10.1), a lytic bone lesion in the left iliac bone (SUVmax 3.7) and muscular metastasis in the deltoid (SUVmax 13.2) and supraspinous (SUVmax 10.4) right muscles. Bone scintigraphy showed no blastic bone metastasis. Breast magnetic resonance imaging was not performed due to the FDG-PET result. Ca15.3 was normal.

In conclusion, it was an LCNEC of the breast, luminal B-like, HER2 negative and according to the 8^th^ edition of the American Joint Committee on Cancer, it was a stage IV tumor. The case was discussed in the Multidisciplinary Tumor Board (MTB). As it was an oligometastatic disease in a very symptomatic patient and the biopsy showed a high Ki67, it was decided to proceed with chemotherapy. The patient started chemotherapy with weekly Paclitaxel. She completed 12 cycles with good tolerance and no significant toxicities.

A reevaluation of FDG-PET was performed, showing a reduction in the size and metabolic activity of the breast and axillary disease and a complete response of the bone and muscular metastasis.

The case was discussed in MTB, and considering the radical aim of the treatment in an oligometastatic disease and respecting the patient’s will, it was decided to proceed with breast surgery. The patient was submitted to modified radical right mastectomy and bilateral oophorectomy. The surgical specimen showed a complete response in the breast, with an inflammatory infiltrate and 7 metastasized lymph nodes out of a total of 22 removed. The pathological staging was ypT0 N2a R0, stage III.

The case was rediscussed in MTB, and it was decided to complete radical treatment with radiation therapy to the right thoracic wall and lymph nodes and to initiate ET associated with a CDK4/6i. After radiation therapy, an aromatase inhibitor and a CDK4/6i were started. The patient is taking the medication with good tolerance, presenting only grade 2 neutropenia and no need for dose adjustments or delays. She has no symptoms, and her last follow-up FDG-PET, 19 months after ET and CDK4/6i, showed no signs of malignant disease.

## DISCUSSION

When considering the diagnosis of NEBC, cellular morphologic features helped distinguish it from a small cell carcinoma, considering their similar immunoprofile. The expression of HR favored the diagnosis of a primary tumor instead of breast metastatic disease. The initial FDG-PET findings also support NEBC diagnosis.

NEBC is currently treated as any invasive breast carcinoma not otherwise specified.^7-12^ Considering it was an oligometastatic disease (three lesions) at presentation, the patient’s symptoms, and the high Ki67, it was decided to start treatment with chemotherapy. Given the response of the primary tumor and the complete metabolic response of all of the metastasis, it was decided to proceed with breast surgery. The pathology report confirmed the complete response of the primary tumor and 7 out of 22 metastasized lymph nodes. Radiation therapy was then performed.

Since the tumor presents a luminal phenotype and taking into account the proven benefit from using CDK4/6i both in adjuvant and in the metastatic setting, it was decided to start ET and a CDK 4/6i. The adequate duration of the CDK 4/6i in this case is debatable.

## CONCLUSION

Reporting cases of NEBC is essential, considering its lower incidence when compared to other breast cancer histology. Thus, publications of these cases should be encouraged in the case report format or case series.

NEBC is a rare entity with scarce evidence to guide treatment. Although NEBC has a worse prognosis than invasive breast carcinoma without neuroendocrine differentiation, a radical treatment should be considered when it presents as an oligometastatic disease. Although not representing enough evidence, as documented by previous reports, this case seems to support the use of CDK4/6i in NEBC.

In addition to the prognosis and the best management of this specific tumor subtype, several other questions are still being studied and debated in the field of breast cancer, such as the role of surgery to primary in metastatic disease and the optimal duration of CDK4/6i.
